# Hypoglossal Schwannoma Masquerading as a Carotid Body Tumor

**DOI:** 10.1155/2012/842761

**Published:** 2012-11-19

**Authors:** Matthew K. Lee, Douglas R. Sidell, Abie H. Mendelsohn, Keith E. Blackwell

**Affiliations:** ^1^Division of Head and Neck Surgery, David Geffen School of Medicine at UCLA, 10833 Le Conte Avenue, CHS 62-132, Los Angeles, CA 90095-1624, USA; ^2^UCLA Department of Head and Neck Surgery, 10833 Le Conte Avenue, CHS 62-132, Los Angeles, CA 90095-1624, USA

## Abstract

*Study Objective*. To describe the clinical presentation, evaluation, and treatment of a hypoglossal schwannoma. *Methods*. We report an unusual case of a hypoglossal schwannoma presenting as a pulsatile level II neck mass at the bifurcation of the external and internal carotid arteries, mimicking a carotid body tumor. Radiologic findings are reviewed in detail. *Results*. A 59-year-old female presented to a tertiary care medical center with complaints of a pulsatile right-sided neck mass. An MRA of the neck was obtained demonstrating a 5 cm mass located at the carotid artery bifurcation and causing splaying of the internal and external carotids. Based on clinical presentation and imaging, a diagnosis of a carotid body tumor was conferred and the patient scheduled for excision. Intraoperatively, the mass was noted to arise from the hypoglossal nerve, remaining independent of the carotid artery. On histopathologic analysis, the mass was determined to be consistent with hypoglossal schwannoma. *Conclusion*. Though rare, the hypoglossal schwannoma should remain a consideration in the evaluation of a parapharyngeal space mass. As this report demonstrates, the clinical and radiologic presentation of a hypoglossal schwannoma may closely mimic that of the more common carotid body tumor.

## 1. Introduction

Magnetic resonance imaging (MRI) and computed tomography (CT) scanning play a significant role in the diagnostic workup of masses located in the retrostyloid parapharyngeal space. Retrostyloid neoplasms frequently arise from neural crest origin, and both paragangliomas and schwannomas are common considerations in the differential diagnosis of such masses. Although paragangliomas and schwannomas share several similarities on imaging, it is often the unique characteristics of each lesion that allow for a definitive radiologic diagnosis. 

The carotid body tumor is the most frequently identified paraganglioma in the head and neck and has a typical appearance on imaging that is rarely replicated by other lesions [1]. Despite this distinctive appearance, other lesions have been known to imitate the appearance of the carotid body tumor, thereby resulting in an inaccurate diagnosis. In this report, we present a patient with a rare hypoglossal schwannoma mimicking a carotid body tumor. To our knowledge, this is the first report of such a lesion. The radiologic and intraoperative characteristics of this lesion are reviewed, and a detailed literature review of relevant parapharyngeal space neoplasms is performed. 

## 2. Case Report

**Figure 1:**
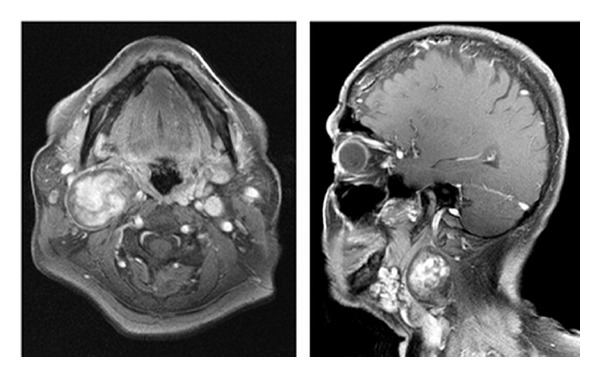
Postcontrast MRI of the neck demonstrating a mass at the level of the carotid bifurcation.

**Figure 2:**
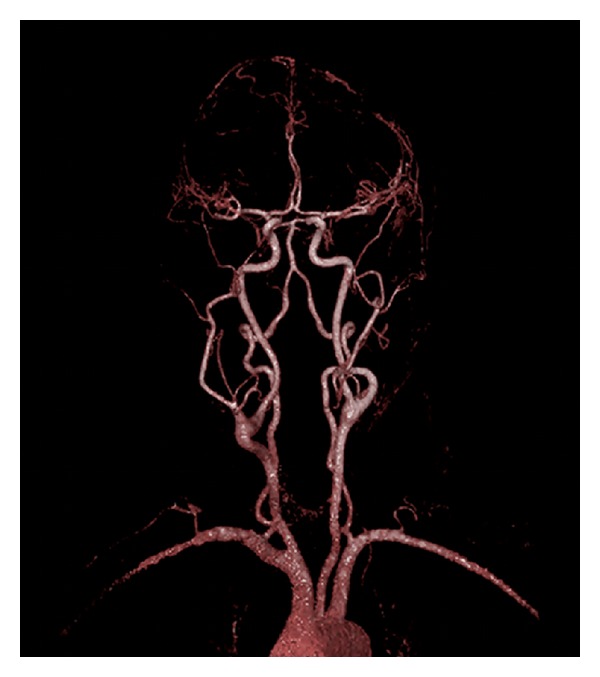
MRA of the neck demonstrating splaying of the internal and external carotid arteries, that is, the “lyre sign.”

A 59 year-old female presented to a tertiary-care university medical center with a two-year history of a progressively enlarging right-sided neck mass. Symptoms on presentation were limited to neck discomfort and mild dysphagia. The patient had an otherwise unremarkable past medical history. On physical examination, a large, mobile, pulsatile level II neck mass was identified. A comprehensive cranial nerve examination was normal. An MRI of the neck demonstrated a mass at the level of the carotid bifurcation, which on postgadolinium imaging demonstrated heterogeneity consistent with internal flow voids (Figure 1). An MRA of the neck was subsequently obtained, demonstrating a 5.3 × 3.3 cm heterogeneous mass causing splaying of the internal and external carotid arteries (Figure 2). 

**Figure 3:**
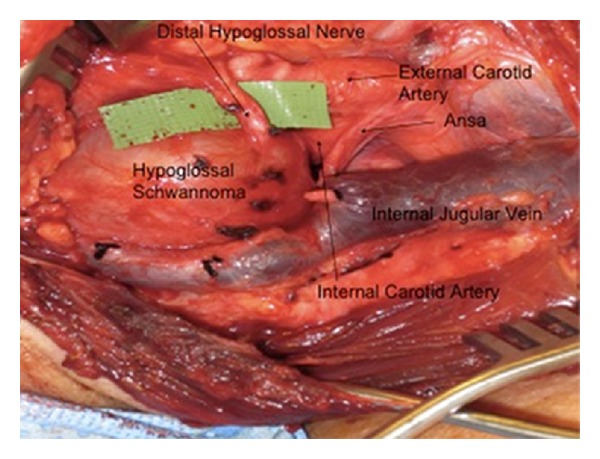
Intraoperative photograph demonstrating a mass originating from the proximal segment of hypoglossal nerve, with the distal segment clearly seen exiting the mass.

Given the radiographic characteristics of the mass and findings on physical examination, a preliminary diagnosis of a carotid body tumor was established. The patient was subsequently brought to the operating suite for a definitive surgical excision. Intraoperatively, a grey, gelatinous-appearing mass was identified at the carotid bifurcation. Upon further dissection, the tumor was unequivocally identified as originating from the proximal segment of hypoglossal nerve, independent of the carotid artery. The distal segment of the hypoglossal nerve was identified as it exited the mass (Figure 3). In light of the preoperative imaging and intraoperative appearance, the constellation of findings favored the diagnosis of hypoglossal schwannoma with inferior extension. Electric stimulation of the mass resulted in a contractile response in the distribution of the hypoglossal nerve, lending credence to this diagnosis.

Initial attempts at subcapsular dissection with preservation of the hypoglossal nerve proved unsuccessful, as fibers of the nerve became progressively attenuated and inseparable from the tumor. The decision was thus made to transect the hypoglossal nerve and excise the mass. After excision, a neurorrhaphy was performed via primary anastomosis using epineural 8-0 nylon sutures in simple interrupted fashion. Final surgical pathology confirmed the working diagnosis of a hypoglossal schwannoma. 

## 3. Discussion

Carotid body paragangliomas are uncommon parapharyngeal space neoplasms that arise from chemoreceptive and sustentacular cells at the carotid bifurcation. These benign lesions display an insidious growth pattern and have been suggested to have an increased incidence in populations living at high altitudes, speaking to the chemoregulatory function of the carotid body [1, 2]. Similar to paragangliomas, head and neck schwannomas are also benign neoplasms with an insidious growth pattern. They are found to be associated with the vestibular nerve in greater than 90% of cases [3, 4]. Although rare, solitary extracranial cervical schwannomas involving the facial, vagus, hypoglossal, and lingual nerve have also been described. Thus, both paragangliomas and schwannomas are frequently included in the differential diagnosis of parapharyngeal space neoplasms [3–5].

 The differential diagnosis for masses located in the parapharyngeal space can be refined based on preoperative imaging characteristics and physical examination findings. Although paragangliomas and schwannomas are both lesions of neural crest origin potentially located in the retrostyloid space, the unique pathophysiology, tumor biology, and management considerations for each lesion make an accurate diagnosis of significant importance. Currently, a standard component of the diagnosis and workup for parapharyngeal space lesions includes MR or CT angiography. At many institutions, formal angiography is only obtained preoperatively based on CTA or MRA findings, with subsequent embolization performed for highly vascular lesions. Although digital and conventional angiography modalities are of great utility, they are not universally available, and conventional MRI and CT play a significant role in the initial diagnosis of these lesions. In the classic 1988 description by Som and colleagues, the use of CT and MR imaging is presented in its ability to narrow the diagnosis of parapharyngeal space lesions based on fat planes, signal characteristics, and the displacement of the carotid artery [6]. These features, although theoretically simple in principle, are important considerations in the evaluation of all parapharyngeal space lesions. Specifically, prestyloid masses, commonly of salivary origin, are known to displace the carotid artery and jugular vein posteriorly. Conversely, retrostyloid lesions such as paragangliomas and schwannomas, displace the vascular axis anteriorly as they enlarge. Evaluation of fat planes is important in differentiating salivary from extrasalivary neoplasms, and the differences in MRI signal characteristics can further improve diagnostic accuracy for certain parapharyngeal space lesions [3, 6]. 

Despite the efficacy of CT and MRI in establishing a diagnosis for typical parapharyngeal space lesions, several imaging similarities between paragangliomas and schwannomas exist that can be confounding. Both schwannomas and paragangliomas are benign retrostyloid lesions that may enhance avidly on MRI following gadolinium infusion, and both have variable signal intensity on T2-weighted images. Imaging characteristics that are distinct to each lesion are therefore employed to assist with establishing a diagnosis. Imaging principles frequently rely on the characteristic blood flow expected for each lesion, or on the anatomic origin of the neoplasm in question [1, 7]. For instance, increased vascularity is often characteristic of paragangliomas and may produce a “salt and pepper” appearance on T2-weighted MRI owing to flow voids within the lesion. The site of origin of the paraganglioma can further aid in distinguishing lesion subtypes, thereby differentiating jugulotympanic lesions of the high skull base or middle ear cleft from those originating from the vagal ganglia or the carotid body. Possibly the most characteristic of such imaging findings is the “lyre sign” produced by the carotid body paraganglioma, as it splays the bifurcation of the internal and external carotid arteries with growth; a finding that is rarely replicated by other lesions [1, 3]. 

 In a similar fashion to that of paragangliomas, the origin of a cervical schwannoma can also be predicted based on the displacement of the great vessels seen on imaging. Schwannomas arising from the sympathetic chain characteristically displace great vessels anteriorly, while vagal schwannomas are known to separate the carotid artery from the internal jugular vein. Large paragangliomas and those arising from the nodose ganglion, however, may occupy the same anatomic location typical to some schwannomas, and therefore share a similar appearance on noncontrast imaging. In this regard, the importance of contrast enhancement becomes increasingly valid. Because only 1/3 of schwannomas are found to enhance with equal avidity to that of paragangliomas and few produce a similar “salt and pepper” appearance on T2-weighted MRI, a diagnosis can still be accurately made under most circumstances [1, 5, 7]. Unfortunately, exceptions to this generalization exist. Specifically, variable regions of necrosis and heterogeneous tumor density of some schwannomas may be difficult to distinguish from the vascular flow voids characteristic of the paraganglioma. Conversely this classic “salt and pepper” appearance may be absent in small paragangliomas and those of limited vascularity, allowing them to mimic schwannomas on imaging [2, 6, 8].

 In the patient presented here, a diagnosis of carotid paraganglioma was established preoperatively based on the presence of enhancement with contrast infusion, apparent large internal vascular flow voids, and the splaying of the internal and external carotid artery on MRI. Despite the preoperative imaging characteristics typical of a carotid body paraganglioma, the lesion was indeed determined to be originating from the hypoglossal nerve intraoperatively. In retrospect, the heterogeneous appearance of the lesion misconstrued as vascular flow voids was likely secondary to variable density of the enlarging neoplasm. Considering the natural location of the hypoglossal nerve, inferior growth of this neoplasm also allowed for the internal and external carotid artery to splay as it further occupied the carotid bifurcation, resulting in the “lyre sign” typical of a carotid body tumor. To our knowledge, this is the first hypoglossal schwannoma mimicking a carotid body paraganglioma to be described in the literature. 

Hypoglossal schwannomas are thought to represent only 5% of all nonvestibular schwannomas, with approximately 110 cases reported in the English literature to date. Of these cases, the vast majority of cases originate intracranially, with or without extracranial extension [5, 7]. Schwannomas arising exclusively from the extracranial segment of the hypoglossal nerve are exceedingly rare. The first such lesion was described by Danely P. Slaughter in 1949 [5]. To our knowledge, less than 40 extracranial hypoglossal schwannomas have subsequently been reported to date. In an exhaustive review of the literature performed by Cavalcanti and colleagues in their 2011 report, only 36 peripheral extracranial hypoglossal schwannomas were reported [9]. Due to the rarity of the extracranial hypoglossal schwannoma, meaningful epidemiological data does not exist. However, when taking into account both intracranial and peripheral lesions, hypoglossal schwannomas appear to occur with a 2.5x female predilection and mean age at diagnosis of 46 years [4, 7]. Hypoglossal paresis has been cited as a potential early symptom on presentation, with an incidence of palsy ranging from 25 to 94% depending on tumor location [4, 5, 7, 10]. Hypoglossal nerve dysfunction is thought to arise secondary to expansion and stretching of nerve fibers during tumor growth. In patients presenting without hypoglossal paresis, slow growth of the lesion and/or abundant vascular supply to the tumor has been cited [7, 10]. Despite the presence or absence of hypoglossal deficits discovered on physical examination, the vast majority of lesions remain asymptomatic for long periods, owing to the slow growth and anatomic location of these neoplasms. 

Treatment for both the carotid body paraganglioma and the hypoglossal schwannoma is primarily surgical. Resection of a cranial nerve schwannoma requires dissection from the nerve fascicles, and complete removal is attempted when possible. Intraoperative monitoring of the hypoglossal nerve has been described as a reliable technique; however monitoring is not a routine procedure at most centers. If sacrifice of the nerve is required for complete excision, great auricular nerve cable graft may be utilized if a tension-free anastomosis is not otherwise possible [5, 7, 10].

## 4. Conclusion

While majority of parapharyngeal space neoplasms are amendable to diagnosis with a high level of certainty based on preoperative patient history and imaging characteristics, circumstances exist where standard diagnostic techniques may be misleading. As this report demonstrates, the clinical and radiologic presentation of a hypoglossal schwannoma may closely mimic that of the more common carotid body tumor. As such, a high level of suspicion must be maintained whenever an aberrancy in the usual presentation of a carotid body tumor is encountered. Though rare, the hypoglossal schwannoma should remain a consideration when evaluating any patient with a mass in the parapharyngeal space.
